# Ⅳ期肺腺癌二线EGFR-TKIs治疗失败后预后因素分析

**DOI:** 10.3779/j.issn.1009-3419.2013.01.07

**Published:** 2013-01-20

**Authors:** 淑珺 戴, 友如 刘, 琳 王, 宝惠 韩, 丽岩 姜

**Affiliations:** 200030 上海，上海交通大学附属胸科医院肺内科 Department of Pulmonary, Chest Hospital Affiliated with Shanghai Jiaotong University, Shanghai 200030, China

**Keywords:** 肺肿瘤, 靶向药物治疗, 预后, Lung neoplasms, Targeted therapy, Prognosis

## Abstract

**背景与目的:**

表皮生长因子受体酪氨酸激酶抑制剂（epidermal growth factor receptor-tyrosine kinases inhibitors, EGFR-TKIs）普遍应用后，其治疗失败后预后情况引起学界的关注。本研究旨在回顾性分析Ⅳ期肺腺癌患者的临床资料，探讨EGFR-TKIs治疗失败后影响预后的相关因素。

**方法:**

收集2009年1月-2012年2月体能状态评分（performance status, PS）为0分-2分的Ⅳ期肺腺癌患者，随访至死亡。主要观察指标为EGFR-TKIs治疗失败后的生存时间（overall survival, OS）。

**结果:**

共81例患者入组，中位OS为9.6个月（Q_L_-Q_U_: 5.4-19.2）。单因素分析显示，PS评分、多部位的转移及靶向药物治疗后出现中/大量胸水对预后有影响（*P* < 0.05），靶向治疗失败后血CEA水平正常及曾有手术史也表明患者更能得到生存时间获益的趋势。多因素分析显示，PS评分、多部位转移及靶向药物治疗后出现中/大量胸水可以作为Ⅳ期肺腺癌患者独立的预后因素（*P* < 0.05）。

**结论:**

晚期肺腺癌PS评分为0分-1分、单脏器的转移及靶向药物治疗后出现无或少量胸水可获得较好的生存时间，应采取积极的化学药物治疗。

## Abstract (en)

**Background and objective:**

The prognostic factors and salvage therapy after the failure of epidermal growth factor receptor-tyrosine kinase inhibitors (EGFR-TKIs) therapy have brought concerns. This study aims to analyze retrospectively the clinical data of patients with advanced lung adenocarcinoma and explore their prognostic factors.

**Methods:**

Patients with integral clinic dates and staged Ⅳ lung adenocarcinoma with performance status (PS) scores from 0 to 2 were enrolled between January 2009 and February 2012 and followed up until death. The primary endpoint was survival time after the failure of EGFR-TKI therapy.

**Results:**

A total of 81 patients were enrolled into the study, and the median overall survival time was 9.6 months (Q_L_-Q_U_: 5.4-19.2). Univariate analysis showed that PS score, metastatic status, and the presence of plural effusion were significantly correlated with patient survival time (*P* < 0.05), whereas normal levels of carcinoembryonic antigen after EGFR-TKI therapy and history of operation showed a trend towards longer survival time. Multivariate analysis showed that the PS score, metastatic status, and plural effusion are independent prognostic factors for advanced adenocarcinoma after the failure of targeted therapy (*P* < 0.05).

**Conclusion:**

A PS score from 0 to 1, single metastasis, and none or less plural effusion may attribute to the good outcome of stage Ⅳ lung adenocarcinoma and should further undergo chemotherapy.

肺癌是危害人类健康的主要恶性疾病之一，也是导致癌症死亡的首要原因，近年来肺癌的发病率逐渐升高，尤其是肺腺癌患者^[1]^，多数患者确诊时病情已属晚期。目前晚期非小细胞肺癌（non-small cell lung cancer, NSCLC）的主要治疗方法为包括化疗和靶向药物在内的综合治疗。在二线或三线治疗中小分子靶向药物表皮生长因子受体酪氨酸激酶抑制剂（epidermal growth factor receptor-tyrosine kinase inhibitors, EGFR-TKIs）吉非替尼（gefitinib）和厄洛替尼（erlotinib）已成为标准治疗之一^[2-4]^，而对有*EGFR*突变的患者EGFR-TKIs已经是一线治疗方案^[5-8]^。然而大多数靶向药物治疗后病情进展或产生耐药后，肺癌患者的预后判断和治疗成为当下的热点。靶向药物治疗失败后，特别是晚期患者的预后情况怎样，到底哪些因素是决定预后的关键，这些问题目前尚无定论。本研究旨在通过回顾性分析2009年1月-2012年2月上海胸科医院收治的Ⅳ期肺腺癌患者的临床特征和随访资料，以探讨其生存时间及影响生存的预后相关因素，以期为临床决策以及预后判断提供参考依据。

## 1 材料与方法

### 1.1 研究对象

2009年1月-2012年2月上海胸科医院收治的经组织或细胞学诊断的肺腺癌患者，均接受过一线化疗和二线靶向药物治疗，靶向药物治疗失败后均采用培美曲塞0.5 g/m^2^，单药或联合奥沙利铂75 mg/m^2^。所有患者均符合以下标准：①经组织或细胞病理学证实为肺腺癌; ②既往曾接受一线含铂化疗和二线吉非替尼或厄洛替尼治疗且病情进展或耐药; ③根据国际抗癌联盟（Union for International Cancer Control, UICC）第七版NSCLC TNM分期确定靶向药物治疗前和治疗后均为Ⅳ期的肺腺癌患者; ④靶向药物治疗失败后共接受至少1次培美曲塞或培美曲塞联合奥沙利铂治疗; ⑤靶向药物治疗失败后复查血常规及肝肾功能等指标基本正常，无严重心脏病和其它合并症; ⑥一线未接受过培美曲塞化疗。EGFR-TKI治疗失败判断标准参照实体瘤疗效评价标准（Response Evaluation Criteria in Solid Tumors, RECIST 1.1）病灶进展（progressivedisease, PD）的判断标准。

### 1.2 方法

末次随访时间为2012年7月20日。随访内容为患者的临床资料，包括年龄、TNM分期、体力状况、吸烟状况、转移部位、接受治疗情况及疗效。主要观察指标为患者靶向药物治疗失败后的生存时间。其中血癌胚抗原（carcinoembryonic antigen, CEA）检测方法：靶向药物治疗失败后至挽救性治疗前，采集患者空腹静脉血4 mL，采用Luminax仪器用电化学发光免疫法检测血清CEA水平，严格按仪器和试剂说明书操作，以10.0 ng/mL为正常值上限。随访时间为从靶向药物治疗失败后化疗的开始时间到患者死亡日期。

### 1.3 统计分析

应用SPSS 16.0统计分析软件，生存分析采用*Kaplan-Meier*法进行*Log-rank*检验。采用单因素分析及*Cox*模型进行预后分析。*P* < 0.05为差异有统计学意义。

## 2 结果

### 2.1 随访及生存情况

2009年1月-2012年2月共有完整随访资料的患者81例，其中一线含铂化疗为多西紫杉醇的患者3例，吉西他滨的患者30例，长春瑞滨的患者32例，紫杉醇类的患者16例。挽救性治疗使用单药培美曲塞的患者46例，使用培美曲塞联合奥沙利铂治疗的患者35例。单药组76.2%（32/42）和联合组78.8%（26/33）的进展后患者接受进一步的化疗或/和靶向治疗。靶向药物治疗失败后患者的中位生存期为9.6个月（Q_L_-Q_U_: 5.4-19.2）（图 1A）。单药和联合组在生存率方面差异无统计学意义（*P*=0.688）。

**1:**
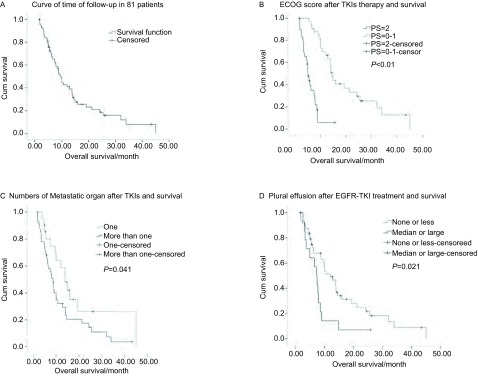
81例患者生存分析（A）以及ECOG PS评分（B）、转移情况（C）、TKI治疗后胸水量（D）与生存关系的多因素分析。 Survival curve of 81 patients (A), multivariate analysis shows PS score (B), situation of metastasis (C), and volume of plural effusion after EGFR-TKI (D) are independent prognostic factors.

### 2.2 单因素分析

对性别、年龄、治疗前体能状态评分（performance status, PS）、手术史、吸烟、靶向药物治疗失败后原发灶增大或出现其它部分转移、放疗、EGFR-TKI的无进展生存时间（progression-free survival, PFS）、EGFR-TKI治疗失败后血CEA升高情况、EGFR-TKI治疗失败后胸水情况、转移器官数目、EGFR-TKI治疗失败后挽救性治疗单药与联合情况进行单因素分析结果见表 1。PS评分为0分-1分和PS评分为2分的患者中位生存时间分别为14.6个月（Q_L_-Q_U_: 10.00-32.03）和5.0个月（Q_L_-Q_U_: 3.38-7.89），两者差异有统计学意义（*P* < 0.05，图 1B）。单个脏器转移的患者和多个脏器转移患者的中位随访时间分别为13.8个月（Q_L_-Q_U_: 7.46-45.01）和8.6个月（Q_L_-Q_U_: 4.90-14.03），两者差异有统计学意义（*P*=0.041，图 1C）。中/大量胸水及少量或无胸水患者的中位随访时间分别为7.3个月（Q_L_-Q_U_: 3.71-8.61）和11.9个月（Q_L_-Q_U_: 5.49-24.21），两者差异有统计学意义（*P*=0.021，图 1D）。

**1:** 81例挽救性治疗的Ⅳ期肺腺癌患者靶向药物治疗失败后生存时间的单因素及多因素分析 Single-factor and multivariate analysis of follow up time for 81 phase IV lung adenocarcinoma patients receiving salvage therapy

Variable	*n*	Median follow up time (month)	Single-factor analysis			Multivariate analysis		
			χ^2^	*P*		HR	95%CI	*P*
Gender			1.228	0.268		1.530	0.652-3.601	0.388
Male	38	8.8						
Female	43	9.9						
Age (year)			0.003	0.956		1.040	0.530-2.042	0.651
< 60	43	9.9						
≥60	38	9.0						
Smoking history			3.136	0.077		1.418	0.512-3.923	0.097
Yes	32	8.8						
No	49	9.9						
ECOG PS score			36.500	0.001		0.180	0.060-0.250	< 0.01
0-1	42	14.6						
2	39	5.0						
Surgery history			2.888	0.089		0.738	0.314-1.737	0.218
Yes	32	12.8						
No	49	8.8						
Radiotherapy history			1.147	0.284		1.473	0.754-2.879	0.116
Yes	39	8.8						
No	42	12.0						
Situation of metastasis			4.100	0.041		0.490	0.280-0.870	0.020
One	36	13.8						
More than one	45	8.6						
PFS with EGFR-TKIs (month)			1.565	0.211		0.772	0.407-1.466	0.078
< 6	40	10.0						
≥6	41	7.9						
Progression situation of TKIs therapy			0.021	0.884		1.589	0.763-3.311	0.884
Enlargement of primary lesion	42	8.8						
New lesion of metastasis	39	9.5						
Plural effusion of post-TKIs therapy			5.293	0.021		0.540	0.310-0.980	0.030
None or less	49	11.9						
Median or large	32	7.3						
CEA value of post-target therapy			0.508	0.476		1.588	0.778-3.241	0.407
Normal	29	10.6						
Abnormal	34	9.0						
Salvage therapy			0.162	0.688		0.998	0.517-1.982	0.278
Pemetrexed alone	46	7.7						
Combined with oxaliplatin	35	10.0						
PS: performance status; PFS: progression free survival								

### 2.3 多因素预后分析

多因素分析显示ECOG评分（0分-1分）（*P* < 0.01, HR=0.18, 95%CI: 0.06-0.25）、远处转移部位数目（*P*=0.02, HR=0.49, 95%CI: 0.28-0.87）及有无中/大量胸腔积液（*P*=0.03, HR=0.54, 95%CI: 0.31-0.98）为其独立预后因素。

## 3 讨论

靶向药物的面世改善了患者的生活质量，提高了患者的无进展生存期。但绝大部分初始对TKI治疗有效的患者在中位缓解7个月-9个月后疾病再次进展，出现对TKI耐药^[9-11]^。对靶向药物治疗失败后的挽救性治疗成为了目前研究的热点。然而靶向药物治疗后疾病进展或产生耐药后，患者的生存时间及预后因素尚无报告。由于患者诊治时间较长，影响预后的因素较复杂，为避免对生存时间有较大的影响，本研究均选用Ⅳ期肺腺癌患者，并着重分析临床上较容易收集的信息进行回顾性分析，简化分层因素对预后的影响，提高生存时间的准确性。本研究共对81例Ⅳ期肺腺癌患者的临床资料和预后因素进行较为全面的分析，结果显示靶向药物治疗失败后的患者中位生存期为9.6个月（Q_L_-Q_U_: 5.4-19.2），同文献^[12, 13]^报道的Ⅳ期肺癌生存时间为8个月-10个月相近。原因可能是本研究选用的都是腺癌患者，并且在后续治疗中培美曲塞对腺癌患者效果较好^[14]^，此外靶向药物治疗副作用较为温和，患者耐受性较好，并且口服靶向药物治疗的患者在家庭经济条件上能更多的采取积极治疗和支持治疗等诸多因素也有关系。

PS评分是目前公认的肺癌患者的一项预后因素。本组无论单因素还是多因素回归分析均显示PS评分为独立预后因素，表明此组数据具有较好的可靠性和准确性。本研究亦得出PS评分是靶向药物治疗失败后生存时间的独立影响因素，PS评分为0分-1分的患者生存时间明显延长（*P* < 0.05），可能与患者的体能状况好，能够采取积极的治疗方案，能耐受化疗的毒副作用有关。

本研究中EGFR-TKI治疗失败原因有肺或其它脏器新发转移病灶，或者是原发病灶或者转移灶增大所致，这两者对晚期肺腺癌患者的中位生存时间影响不大，然而EGFR-TKI治疗失败后存在中/大量的胸腔积液与无或少量胸腔积液的患者中位生存时间有统计学上的差异。原因可能与中/大量胸腔积液往往伴随着患者的低营养状态、低PS评分，有效的治疗方法难以更直接起效等有密切的关系，患者出现脑或者骨转移时可通过放疗或者相应的措施延缓病情进展，而当患者出现新发胸腔积液时则会使患者产生胸闷等临床症状，引流胸水则易造成患者体内蛋白水平下降，在思想上加重患者的负担以及身体上引起患者的不适，致使体质衰弱，对后续的治疗也带来影响。

单因素分析提示手术治疗史具有影响预后的趋势。原因可能为手术治疗减轻了肿瘤负荷，肿瘤异质性低于初诊的进展期肺癌，因此在挽救性治疗中疾病相对容易得到控制，然而在多因素分析中，手术史并不能作为一个独立的预后因素用来评判患者的生存预期。血清CEA是最早发现的肺癌肿瘤标志物，在腺癌中升高较明显，被广泛地应用于肺腺癌的临床诊断与治疗分析中，有学者提出在早期肺腺癌患者治疗前的血清CEA水平能够用于预测微转移，术前CEA阳性患者复发、转移以及死亡率均高于阴性组, 在预测治疗疗效中有一定的意义^[15, 16]^。本研究晚期肺腺癌患者生存因素中，CEA水平无论是单因素分析还是多因素分析，均不能作为一个预后因素用来评判晚期肺腺癌的生存情况，差异没有统计学意义。

文献报道*EGFR*和*KRAS*基因状态对患者预后也有影响，但由于本研究入选的患者大多由细胞病理学明确诊断，且部分术后复发患者亦未常规组织进行EGFR表达或突变的检查，分子水平的差异影响患者生存时间需在今后的研究中加以论证。但由于病例入选条件的限制较多，研究及样本量相对偏小，需更大样本的研究及前瞻性的临床研究以提高结果的可信度。

总之，二线TKI靶向药物治疗失败后患者仍可获得较好的生存时间，ECOG PS评分较好、转移部位单一、无或者少量胸水的患者的生存时间较长，应积极采取挽救性治疗及支持治疗。
